# Effectiveness of Maternal Respiratory Syncytial Virus Vaccination in Conferring Infant Immunity: Review and Future Perspectives

**DOI:** 10.3390/vaccines14030232

**Published:** 2026-02-28

**Authors:** Masatoki Kaneko, Junsuke Muraoka

**Affiliations:** 1 Department of Obstetrics and Gynecology, Faculty of Medicine, University of Miyazaki, Miyazaki 889-1692, Japan; jyunsuke_muraoka@med.miyazaki-u.ac.jp; 2 Graduate School of Nursing Science, Faculty of Medicine, University of Miyazaki, Miyazaki 889-1692, Japan

**Keywords:** respiratory syncytial virus, fusion protein, prefusion F protein, antigenic evolution, neutralizing antibodies, maternal immunization, placental antibody transfer, monoclonal antibodies, infant protection, vaccine development

## Abstract

Respiratory syncytial virus (RSV) is a leading cause of acute lower respiratory tract infection in infants and young children worldwide and continues to impose a substantial disease burden despite recent advances in preventive strategies. Natural infection does not confer durable protective immunity, resulting in repeated reinfections, with the most severe disease occurring during early infancy. This review examines antibody-mediated prevention of RSV infection, with particular emphasis on vaccine development and maternal immunization. We reviewed current evidence on RSV pathogenesis, immune evasion, and antigenic characteristics relevant to vaccine design, focusing on viral surface glycoproteins targeted by preventive strategies. Recent data on licensed vaccines, long-acting monoclonal antibodies, and maternal immunization approaches were also evaluated. The RSV fusion (F) glycoprotein is the principal target of neutralizing antibodies and underpins currently licensed vaccines and monoclonal antibody products. Although circulating RSV strains show gradual antigenic evolution, primarily in the attachment protein, the F protein remains relatively conserved, resulting in only modest reductions in neutralization by human polyclonal sera over time. Constrained evolution of the F protein likely contributes to the sustained effectiveness of F-based interventions. However, waning F-specific neutralizing antibody titers contribute to susceptibility to reinfection, underscoring the importance of passive immunization strategies during early life. Maternal vaccination and long-acting monoclonal antibodies represent key advances in protecting young infants against RSV, but challenges remain in achieving equitable global implementation. Continued evaluation of antigenic evolution, the durability of protection, and optimization of maternal and infant immunization strategies will be critical for long-term disease control.

## 1. Introduction

Respiratory syncytial virus (RSV) is widely distributed globally, infecting more than half of all children during their first RSV season and nearly all by 2 years of age [1,2]. However, these early infections do not confer lifelong immunity. RSV remains the leading viral cause of lower respiratory tract disease in infants and young children, accounting for approximately 50% of pneumonia cases and 50–90% of bronchiolitis cases.

The clinical spectrum of RSV infection ranges from mild, cold-like symptoms to severe lower respiratory tract disease. In infants, RSV most commonly presents as bronchiolitis, characterized by inflammation and obstruction of the small airways. Infants younger than 6 months are at the highest risk of severe disease [3,4,5]. Complications include apnea and acute encephalopathy, and recurrent wheezing is a common long-term sequela. Approximately 2–3% of infants require hospitalization due to RSV infection.

Although high-risk groups include premature infants and children younger than 24 months with immunodeficiency, congenital heart disease, chronic lung disease, or Down syndrome, most severe cases occur in full-term, otherwise healthy infants [6,7,8,9,10,11]. RSV also causes substantial morbidity in older adults, particularly in developed countries [12].

This review provides a comprehensive overview of RSV infection in infants, with emphasis on epidemiology, pathogenesis, maternal immunization, and antibody-mediated protection, including recent advances in maternal vaccines. To enhance methodological transparency, we briefly describe our literature identification approach. We searched PubMed, Embase, and major public health agency repositories using terms such as “RSV,” “maternal immunization,” “RSVpreF,” “nirsevimab,” and “monoclonal antibodies.” English-language human studies and key preclinical findings published between 2010 and 2025 were considered. As this is a narrative review, the search was not systematic; instead, it aimed to capture the most relevant and recent evidence to inform current scientific and policy discussions.

In addition to summarizing available evidence, this review incorporates a critical appraisal of study design limitations, heterogeneity across populations, and potential sources of bias, particularly in real-world effectiveness studies and observational comparative analyses. This analytical perspective is essential for contextualizing emerging data and informing evidence-based policy decisions. Furthermore, we highlight methodological considerations such as confounding, selection bias, and the potential role of target trial emulation as a framework for improving causal inference in future observational studies.

## 2. Epidemiology and Disease Burden

### 2.1. Disease Burden

RSV is one of the leading causes of acute lower respiratory tract infection in children younger than 5 years worldwide. Globally, RSV is estimated to cause approximately 33 million lower respiratory tract infections, 3.6 million hospitalizations, and around 100,000 deaths annually, most of which occur in low- and middle-income countries (LMICs) [13]. More than 90% of severe cases and deaths are reported in countries with limited healthcare resources [13]. RSV also remains a major cause of hospitalization among infants and young children in developed countries. Reports indicate that a substantial proportion of infant respiratory hospitalizations in the United States and European countries, estimated at 57–72%, are attributable to RSV infection [13]. Between 2017 and 2018, 18,220 Japanese children younger than 2 years were diagnosed with RSV infection according to the Japanese Medical Data Center database. Of these, 25% required hospitalization, and 90% of hospitalized children had no known RSV risk factors. Acute respiratory failure occurred in 12% of hospitalized cases. The average hospital stay was approximately 1 week, and 7% of patients required some form of mechanical ventilation. The incidence of RSV-related hospitalization among children younger than 2 years was 23.2 per 1000 person-years, increasing to 35.4% per 1000 person-years among infants younger than 6 months. Extrapolation of data from the Japan Data Management Consortium to the national population suggests that approximately 119,000–138,000 RSV-related infections occur annually among children younger than 2 years in Japan [14]. RSV also imposes a substantial disease burden in adults and older individuals. Some reports indicate that RSV-related mortality rates are higher in individuals aged 70 years and older than in children younger than 5 years [15].

The burden of RSV infection in pregnant women remains poorly defined, largely because few studies have specifically examined this population. Available evidence suggests that RSV infection occurs in a notable proportion of pregnant women, although the true incidence and full clinical spectrum remain uncertain. A systematic review based primarily on data from high-income countries, including Canada, the United States, and several European nations, reported that the incidence of RSV infection among pregnant women ranged from 10% to 13% during the RSV season [16,17]. In contrast, a broader review incorporating data from LMICs found that the proportion of acute respiratory infections in pregnant women attributable to RSV ranged from 0.9% to 10.7%, highlighting substantial geographical and methodological variability in reported estimates [16,17]. These wide ranges underscore the heterogeneity of available studies, which differ in diagnostic methods, surveillance intensity, and population characteristics, limiting direct comparability across settings.

Hospitalization and mortality associated with RSV infection appear to be uncommon among pregnant women, suggesting that most infections are mild or self-limiting. However, severe disease may occasionally occur, particularly in women with underlying comorbidities or pregnancy-related immunological changes. The impact of maternal RSV infection on perinatal outcomes remains uncertain. Some studies have suggested a possible association between maternal RSV infection and adverse pregnancy outcomes, including preterm delivery and low birth weight [18,19], but available data are inconsistent and frequently limited by small sample sizes, retrospective designs, and lack of laboratory confirmation of RSV infection. Given these methodological limitations, current evidence is insufficient to establish a causal relationship, and further prospective studies with standardized diagnostic criteria are needed.

### 2.2. Epidemic Outbreak

The timing and seasonality of RSV epidemics differ between temperate and tropical or subtropical regions. Furthermore, many regions have reported disruptions to traditional seasonal patterns following the implementation of public health measures during the COVID-19 pandemic, including lockdowns, mask use, social distancing, and school closures. In the United States, RSV epidemics traditionally peaked during winter before the COVID-19 pandemic. Since 2021, however, epidemic peaks have shifted earlier, with trends toward larger outbreak waves and infant hospitalization rates exceeding historical levels [20]. Similarly, a European study reported that the 2023–2024 RSV season began earlier than usual and showed greater variability in hospitalization numbers [21]. In Japan, RSV infection peaks were observed in September in both 2018 and 2019; however, since the onset of the COVID-19 pandemic, seasonal patterns have been markedly disrupted, with substantial year-to-year and regional variation making outbreaks difficult to predict [22].

The “immunity debt” hypothesis has been proposed to explain these changes. Reduced exposure to RSV over recent years may have increased the number of susceptible individuals, potentially contributing to the magnitude of the observed resurgence [23,24]. However, empirical evidence supporting this hypothesis remains limited, and alternative explanations—such as changes in testing practices or healthcare-seeking behavior—may also contribute to observed trends. Such fluctuations complicate epidemic forecasting and healthcare resource planning, underscoring the need to strengthen RSV surveillance systems [21].

### 2.3. Shift and Expansion of Age Structure

Recent reports indicate changes in the age distribution of reported RSV infections, with a growing proportion occurring in children aged 2–5 years. For instance, one study found that the proportion of infections in this age group increased from 32.1% during the 2018–2019 season to 51.8% in 2023 [21]. This suggests that RSV, traditionally concentrated in infants and young children, may now be affecting a slightly broader pediatric age range [21]. Furthermore, comparisons across epidemic periods indicate a noticeable rise in RSV infection and hospitalization rates among adults and older adults relative to previous years [15]. These shifts may reflect altered population immunity following the COVID-19 pandemic, but the relative contributions of viral evolution, behavioral changes, and surveillance artifacts remain uncertain. In addition, increased RSV testing and heightened clinical awareness in older children and adults may partially contribute to the apparent rise in reported cases.

## 3. Pathophysiology

### 3.1. Structure of Virus

RSV is an enveloped, non-segmented, negative-strand RNA virus belonging to the family Paramyxoviridae. Its genome consists of 10 genes encoding 11 proteins. Three viral proteins are displayed on the RSV surface: small hydrophobic (SH) protein, fusion (F) glycoprotein, and attachment (G) glycoprotein [25]. The SH protein can serve as a target for antibodies with Fc-mediated effector functions. The F glycoprotein mediates viral fusion with the host cell membrane and exists in two major conformations: prefusion (pre-F) and postfusion (post-F). The metastable pre-F protein undergoes conformational changes to drive membrane fusion, forming the stable post-F structure. The G glycoprotein functions as an attachment protein and is a transmembrane protein anchored at the N terminus, analogous to hemagglutinin (H) and neuraminidase (HN) proteins in other paramyxoviruses. Both F and G proteins are key targets of neutralizing antibodies [26,27], and high antibody titers against these proteins are associated with a reduced risk of RSV lower respiratory tract infection [28]. Although the structural biology of RSV is well characterized, the relative contribution of each viral protein to clinical disease severity remains incompletely understood. Variability in host immune responses and viral genetic diversity may modulate pathogenicity in ways that are not fully captured by current models. In particular, the antigenic sites on the F protein—while relatively conserved—may influence the durability of vaccine-induced immunity, highlighting the importance of continued monitoring for antigenic drift and its potential implications for booster strategies and long-term vaccine policy.

### 3.2. Mechanism of Severe RSV Infection

Postmortem analysis of lungs from fatal RSV cases unaffected by medical intervention likely reflects the virus’s intrinsic capacity to cause severe disease. The RSV antigen is detected in alveolar epithelial cells, while the bronchial lumen contains desquamated and degenerated cells protruding in a polypoid fashion, along with neutrophil infiltration and minimal lymphocytic presence [29,30,31]. These deformed and desquamated cells are thought to represent syncytia formed by the fusion of multiple RSV-infected cells. Similar findings have been observed in vitro in cell and tissue cultures, as well as in vivo animal models [30]. This extensive cellular degeneration is a characteristic of RSV infection and is rarely observed in autopsy findings of other viral respiratory infections. Additionally, increased mucus production and infiltration by inflammatory cells—primarily neutrophils—further narrow the airways. This airway obstruction is particularly critical in infants, whose bronchiolar lumen diameter averages approximately 120 µm, roughly half that of adults, contributing to respiratory distress. However, the extent to which these pathological findings directly correlate with clinical severity in living infants remains uncertain, as postmortem samples may overrepresent the most severe disease phenotypes. Prospective clinical–pathological correlation studies are limited.

### 3.3. Viral Tropism

The replication capacity of RSV is central to understanding the mechanisms underlying severe infection. Viral tropism is influenced by the presence of specific receptors on host cell, the suitability of the intracellular environment, and the virus’s ability to evade host immune responses.

Cellular degeneration, a hallmark of severe RSV disease, results from the activity of multiple viral proteins involved in the replication cycle and is thought to be facilitated by immune evasion mechanisms that promote efficient viral replication and persistence [31]. The G glycoprotein on the RSV envelope mediates initial attachment by binding to airway epithelial cell surface receptors, including CX3CR1 and heparan sulfate proteoglycans. Nucleolin on the cell surface subsequently interacts with the F glycoprotein, triggering fusion between the viral envelope and the host cell membrane. Once inside the cell, RSV genes are transcribed, viral proteins are synthesized using host machinery, and the replicated viral genome is assembled into new virions. These virions either bud from the host cell or, through F-mediated fusion, form syncytia with neighboring cells, contributing to tissue pathology.

The host detects RSV through pattern recognition receptors, including TLR4 (toll-like receptor 4), RIG-I (retinoic acid-inducible gene I), and MDA5 (melanoma differentiation-associated protein 5), which help control infection via local cytokine production and the induction of virus-specific cellular immunity. However, RSV employs immune evasion mechanisms that inhibit type I interferon (IFN) responses [32]. This inhibition is primarily mediated by the nonstructural proteins NS1 and NS2. NS1 interferes with RIG-I binding to MAVS (mitochondrial antiviral signaling protein), thereby suppressing IFN production downstream of the MAVS–RIG-I pathway. NS2 similarly interacts with RIG-I and MDA5, blocking downstream signaling in the IFN synthesis pathway. RSV has also been reported to modulate mitochondrial function, collectively impairing innate immune responses [33]. In vitro studies using an NS2-deficient RSV mutant showed no morphological abnormalities in infected cells. In contrast, parainfluenza virus type 3 engineered to express RSV-derived NS2 induced swollen ciliated cells similar to those observed with RSV [30]. In hamster models, the presence or absence of NS2 expression correlated with the severity of pathological findings, further highlighting NS2’s role in RSV-mediated tissue damage [34].

Other RSV proteins also contribute to viral pathogenicity. The G glycoprotein exists in a secreted form, which has been reported to suppress the activation of innate immune cells and reduce cytokine production [35]. Based on the characteristics of the G protein, RSV is broadly classified into types A and B, with further subdivision into multiple genotypes. Several studies have explored associations between these subtypes and disease severity [25,36]. However, clinical data are inconsistent, and some evidence suggests that circulating strains do not differ significantly in their capacity to cause severe disease [37].

Overall, while substantial progress has been made in elucidating RSV tropism and immune evasion, significant gaps remain in understanding how viral and host factors interact to determine disease severity. These uncertainties highlight the need for integrated clinical, virological, and immunological studies.

## 4. Passive Immunity Against RSV

### 4.1. Surface Proteins of RSV and Antibody Targets

The F glycoprotein is the primary target of neutralizing antibodies, as it is highly conserved across all currently circulating RSV genotypes [38,39,40,41]. The F protein exists in two major conformations: pre-F and post-F. Antibodies directed against the pre-F conformation exhibit particularly potent neutralizing activity. During viral entry, the metastable pre-F form undergoes structural rearrangement to mediate membrane fusion, resulting in the stable post-F conformation.

**Figure 1:**
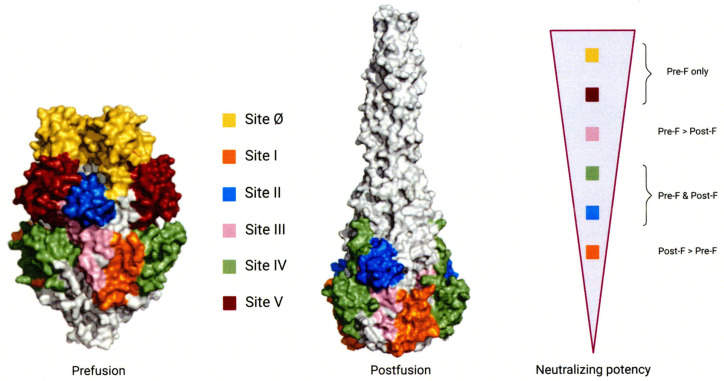
Vaccine-Relevant Antigenic Sites on the RSV F Protein in Prefusion and Postfusion Conformations [38]. Vaccine-Relevant Antigenic Sites on the RSV F Protein in Prefusion and Postfusion Conformations. Trimeric prefusion and postfusion conformations of the respiratory syncytial virus (RSV) F protein, with antigenic sites shown in distinct colors. Antigenic sites II and IV are present in both prefusion and postfusion conformations, whereas sites Ø and V are specific to the prefusion conformation. The right panel illustrates the relative neutralizing potency of antibodies targeting each antigenic site, highlighting their relevance for vaccine design. It should be noted that the observed differences in neutralizing potency may partially reflect variations in antibody affinity or other intrinsic properties of the monoclonal antibodies (mAbs). The figure was created using PyMOL and BioRender.com [38].

Neutralizing antibodies recognize multiple antigenic sites on the F protein, designated as sites Ø and I–V [42]. During the pre-F to post-F transition, sites Ø and V are lost [43,44], making them specific to the pre-F form, whereas sites I–IV are present in both conformations. Binding affinities vary even among shared sites: for example, site III is more accessible on pre-F, whereas site I is more prominent on post-F (Figure 1) [45].

In contrast, the G glycoprotein exhibits greater sequence variability than the F protein [38,39,40,41]. Although the G protein contains multiple neutralization-sensitive epitopes, the F protein’s higher degree of conservation across RSV subgroups A and B makes it a more promising target for preventive interventions. Clinical data indicate that neutralizing antibodies directed against the pre-F conformation correlate with protection in humans [43,46,47,48,49,50,51,52,53,54,55,56]. Accordingly, current vaccine and monoclonal antibody development efforts are focused on eliciting pre-F-specific immune responses to protect infants and other high-risk populations.

Nevertheless, the precise correlates of protection remain incompletely defined, and neutralizing antibody titers alone may not fully capture the complexity of protective immunity. Differences in assay methodology, epitope accessibility, and Fc-mediated effector functions contribute to heterogeneity across studies, underscoring the need for standardized immunogenicity assessments. Waning of F-specific neutralizing antibodies typically occurs over months to years, explaining the lifelong susceptibility to reinfection; however, reinfections in older children and adults are generally milder due to partial immunity from prior exposures. Furthermore, because antigenic sites Ø and V are unique to the pre-F conformation, even subtle antigenic drift affecting these epitopes could influence vaccine-induced immunity and may have implications for long-term vaccine durability and potential booster strategies.

### 4.2. Licensed RSV Vaccines

Most current RSV vaccine candidates are based on the pre-F-stabilized F protein, due to its strong ability to elicit high titers of neutralizing antibodies, its critical role in viral entry, and its high sequence conservation across RSV subgroups [39,40,57,58,59]. While the pre-F-stabilized conformation remains the primary antigenic construct, alternative strategies are under investigation, including vaccines targeting the full-length F protein or selected antigenic sites within the F glycoprotein.

The US Food and Drug Administration (FDA) has approved the first RSV vaccines for clinical use: three for older adults and one for maternal immunization [60]. A brief overview of these vaccines is provided below.

Abrysvo^®^ (RSVpreF), developed by Pfizer, is a bivalent subunit vaccine containing pre-F-stabilized F proteins from both RSV-A and RSV-B subtypes. It is approved for use in older adults and for administration during pregnancy to provide passive immunity to infants [40]. Abrysvo^®^ is currently the only maternal RSV vaccine and does not contain an adjuvant. A phase 2 randomized trial in 406 healthy pregnant women at 24–36 weeks’ gestation evaluated the effect of aluminum hydroxide adjuvantation. Participants received recombinant RSV vaccine at 120 μg or 240 μg, with or without adjuvant, or placebo. In the nonadjuvanted 120-μg group, geometric mean placental transfer ratios were 2.10 (95% confidence interval [CI], 1.78–2.29) for RSV-A and 2.10 (95% CI, 1.75–2.53) for RSV-B, indicating that adjuvantation did not measurably enhance placental antibody transfer [56].

Arexvy^®^ (RSVpreF3), developed by GSK, is a protein subunit vaccine composed of a stabilized trimeric pre-F F glycoprotein formulated with the AS01E adjuvant system to enhance immunogenicity [41]. However, GSK’s maternal unajuvanted vaccine trial test was halted after a numerical imbalance in preterm births was observed, emphasizing the clinical importance of pregnancy-specific safety data for program continuation [61].

mRESVIA (mRNA-1345), developed by Moderna, is an mRNA-based vaccine encoding a membrane-anchored pre-F F glycoprotein derived from an RSV-A strain. This platform leverages mRNA technology to achieve rapid antigen expression and robust neutralizing antibody responses [57].

Together, these approvals represent a major milestone in RSV vaccine development and demonstrate the success of structure-based antigen design focused on the pre-F F protein.

However, differences in antigen presentation, adjuvant use, and platform technology may influence immunogenicity profiles, durability of protection, and safety considerations across populations. Comparative data remain limited, and ongoing postmarketing surveillance will be essential to refine vaccine policy.

## 5. Maternal RSV Pre-F Protein Vaccine

### 5.1. Immunoglobulin G Placental Transfer During Healthy Pregnancy

Low-molecular-weight compounds (<500 Da) cross the placenta readily via passive diffusion. In contrast, maternal immunoglobulin G (IgG), a ~160 kDa protein, is transferred to the fetus through a tightly regulated, receptor-mediated process.

Transplacental IgG transport occurs across the syncytiotrophoblast, which is directly exposed to maternal blood. Maternal IgG is internalized into endosomal compartments, where the neonatal Fc receptor (FcRn) is expressed on the endosomal membrane. Endosomal acidification promotes high-affinity binding of IgG to FcRn, protecting it from lysosomal degradation and enabling directional transcytosis. The IgG–FcRn complex is transported to the fetal-facing basal membrane, where exposure to physiological pH facilitates IgG release into the fetal circulation. When maternal IgG concentrations are high, FcRn-mediated transport can become saturable, leading to increased intracellular degradation of excess IgG [62,63,64,65].

Fetal IgG concentrations generally mirror maternal levels but are limited by the finite capacity of FcRn-mediated transport. Consequently, the efficiency of placental IgG transfer depends largely on FcRn expression and availability, whereas IgG molecules not bound to FcRn are directed to lysosomal degradation [66].

IgG transfer begins early in gestation, with detectable transport as early as 13 weeks, and progressively increases throughout pregnancy, peaking in the third trimester [39]. Longitudinal studies by Malek et al. reported a steady rise in fetal IgG concentrations between 17 and 41 weeks of gestation [67]. During mid-gestation (17–22 weeks), fetal IgG levels represent only 5–10% of maternal concentrations, increasing to approximately 50% by 28–32 weeks. The majority of fetal IgG acquisition occurs in the final weeks of gestation, and at term, fetal IgG concentrations typically exceed maternal levels by 20–30% [68]. Notably, cord blood IgG concentrations rise sharply after 36 weeks of gestation.

Clinical and demographic maternal factors—such as age, body weight, parity, and mode of delivery—appear to have minimal influence on placental IgG transfer [69]. Instead, gestational age is the primary determinant of neonatal IgG concentrations, consistent with evidence that placental FcRn expression increases with advancing gestation and reaches its maximum in late pregnancy.

However, substantial interindividual variability in IgG transfer efficiency has been reported, influenced by maternal infection, hypergammaglobulinemia, placental inflammation, and IgG subclass distribution. These factors may modify the magnitude of vaccine-induced antibody transfer and contribute to heterogeneity observed across clinical studies. Moreover, although FcRn-mediated transport is well characterized mechanistically, the relative contributions of maternal antibody titers, subclass composition, and placental function to infant protection remain incompletely understood. Further research integrating immunologic and clinical outcomes is needed to refine optimal maternal vaccination strategies.

### 5.2. Robust Immune Responses Induced by RSVpreF

Maternal immunization provides a dual benefit: direct protection of pregnant women and passive immunity for infants via transplacental antibody transfer. The efficiency of antibody transfer after maternal vaccination, however, can be influenced by several factors, including gestational age at immunization and delivery, the interval between vaccination and birth, total maternal IgG concentrations, and vaccine-specific IgG and IgG subclass profiles [70,71].

Final analyses from the Maternal Immunization Study for Safety and Efficacy (MATISSE) trial demonstrated that RSVpreF vaccination elicited robust immune responses in pregnant women and achieved efficient transplacental transfer of RSV-specific antibodies to newborns. These outcomes were consistent across clinically relevant subgroups, including variations in gestational age at vaccination and delivery, vaccination-to-delivery interval, geographic region, and maternal age [72].

From a clinical perspective, newborns achieved high combined RSV-A/RSV-B-neutralizing antibody titers across most subgroups, supporting the capacity of maternal RSVpreF immunization to confer early-life passive protection. Infants born ≥14 days after maternal vaccination exhibited particularly high neutralizing titers, with geometric mean ratios relative to placebo of 10.2 for those delivered 14–29 days postvaccination and 12.2 for those delivered ≥30 days postvaccination. These results suggest that a minimum interval of approximately 2 weeks between maternal vaccination and delivery is sufficient to enable effective placental transfer of protective antibodies.

Although infants delivered within 14 days of maternal vaccination had lower neutralizing titers, antibody levels were still 4.1-fold higher than those in infants born to placebo recipients, indicating that partial passive immunity may be conferred even when the interval between vaccination and delivery is short. This finding has important clinical implications for women who deliver earlier than expected after vaccination.

Consistent with these results, maternal-to-fetal placental transfer ratios of combined RSV-A/RSV-B–neutralizing antibodies in the RSVpreF group generally approached or exceeded unity, reflecting efficient antibody transfer. Reduced transfer ratios were observed only in infants born <14 days or 14–29 days after vaccination (0.32 and 0.67, respectively), highlighting the importance of sufficient time for antibody maturation and placental transport. Nevertheless, newborn neutralizing antibody titers in all RSVpreF subgroups remained substantially higher than those in the placebo group.

Despite these encouraging findings, it is important to note that subgroup analyses in MATISSE were exploratory and not powered for formal interaction testing. Therefore, observed differences in antibody titers across gestational windows should be interpreted cautiously, and further studies are needed to clarify the optimal timing of maternal vaccination. Additionally, variability in maternal IgG subclass distribution, placental FcRn expression, and maternal comorbidities may influence the magnitude of antibody transfer, contributing to heterogeneity observed across populations. These factors underscore the need for continued research to refine maternal immunization strategies and to better understand determinants of infant protection.

### 5.3. MATISSE Trial

Recent maternal RSV vaccine development has shifted from mixed results with post-F constructs to encouraging efficacy for pre-F-stabilized F immunogens. The MATISSE trial was a phase 3, multicenter, double-blind, placebo-controlled trial designed to evaluate the safety and efficacy of a bivalent RSV pre-F protein-based (RSVpreF) vaccine (Abrysvo^®^) administered during pregnancy to protect infants against RSV-associated lower respiratory tract illness (LRTI). Conducted across 18 countries, the trial enrolled over 7300 pregnant participants at 24–36 weeks’ gestation who were randomly assigned to receive a single intramuscular dose of RSVpreF or placebo. The vaccine met one of its two primary endpoints, demonstrating efficacy in preventing medically attended severe RSV-associated LRTI in infants. Within the first 90 days after birth, vaccine efficacy reached 81.8%, with substantial protection maintained through 180 days (69.4%). Although the second primary endpoint—prevention of all medically attended RSV-associated LRTI—did not meet the predefined statistical success criterion at 90 days, efficacy estimates still favored the vaccine, showing 57.1% protection at 90 days and 51.3% protection at 180 days. Secondary outcomes further supported the vaccine’s benefit, particularly in reducing RSV-associated hospitalizations, with efficacy of 67.7% through 90 days and 56.8% through 180 days. Exploratory analyses also indicated reduced medically attended RSV-associated respiratory tract illness throughout the first 6 months of life (Table 1) [56].

Safety findings from the MATISSE trial were reassuring. Maternal reactogenicity was generally mild to moderate, with injection site pain being the most frequently reported event. Rates of adverse events, serious adverse events, and adverse events of special interest in both mothers and infants were similar between the vaccine and placebo groups, with no safety signals identified. A small, nonsignificant increase in preterm births was observed in the vaccine group (Table 2) [56]. Although the MATISSE trial enrolled pregnant women from 24 weeks’ gestation, regulatory approvals for Abrysvo^®^ as a maternal vaccine vary: the US FDA licenses its use from 32 weeks, the UK Medicines and Healthcare Products Regulatory Agency from 28 weeks, and both the European Medicines Agency (EMA) and the agency in Japan from 24 weeks of gestation (Table 3) [73,74,75,76].

In addition, the incidence of hypertensive disorders of pregnancy (HDP) among trial participants and placebo recipients was as follows: pre-eclampsia occurred in 68 participants (1.8%) versus 53 placebo recipients (1.4%), hypertension in 13 (0.4%) versus 6 (0.2%), and gestational hypertension in 41 (1.1%) versus 38 (1.0%). Although the rates of HDPs were numerically higher in the vaccine group, no statistically significant differences were observed between the groups.

Importantly, the MATISSE trial was a randomized controlled trial, and therefore concerns such as immortal time bias do not apply to its design. Immortal time bias is relevant primarily to observational postmarketing studies in which exposure classification may be misaligned with the timing of outcome onset. The overall incidence of HDPs in MATISSE was substantially lower than the prevalence reported in general pregnant populations [77], likely reflecting the enrollment of a healthier cohort and the controlled conditions of a clinical trial. Thus, while numerical imbalances warrant continued monitoring, the trial data do not support a causal association between RSVpreF vaccination and HDPs. Furthermore, subgroup analyses—including those evaluating gestational timing—were exploratory and not powered for formal interaction testing. Therefore, differences in safety or efficacy across gestational windows should be interpreted with caution.

Overall, maternal immunization with RSVpreF provided significant protection against severe RSV-associated disease in early infancy, the age group at highest risk for complications, and demonstrated a favorable safety profile. These results support maternal vaccination as an effective strategy to prevent severe RSV illness during the critical first few months of life. Continued postmarketing surveillance remains essential to refine safety estimates, particularly regarding rare outcomes such as HDPs and preterm birth, and to ensure that findings from controlled trials translate reliably to broader, more diverse pregnant populations.

### 5.4. Maternal RSV Vaccination Timing and Effectiveness

Abrysvo^®^ can be administered to pregnant individuals between 24 and 36 weeks of gestation; however, vaccine efficacy varies with timing of administration. Higher levels of protection are achieved when vaccination occurs between 28 and 32 weeks or between 32 and 36 weeks of gestation, compared with administration between 24 and 28 weeks. Specifically, efficacy against severe RSV-related LRTI in infants up to 6 months of age was 43.7% for vaccination at 24–28 weeks, compared with 88.5% for 28–32 weeks and 76.5% for 32–36 weeks. Similarly, reductions in RSV-related LRTI requiring medical care were 20.7% for 24–28 weeks, 67.4% for 28–32 weeks, and 57.3% for 32–36 weeks [56,72,78]. However, these gestational timing subgroup analyses were exploratory and not prespecified in the MATISSE trial. Interaction tests were not statistically significant, and the study was not powered to detect differences between gestational windows. Therefore, the apparent superiority of vaccination at 28–32 or 32–36 weeks should be interpreted with caution. Based on these findings, several countries and regions recommend maternal vaccination during the 28–36-week or 32–36-week gestational window to optimize antibody transfer and confer maximum protection to infants (Table 3) [75,76,79,80,81,82,83,84,85,86,87].

Two detailed reports highlight the timing and effectiveness of maternal RSV vaccination.

In the United States, the FDA recommends immunization of pregnant individuals at 32–36 weeks of gestation. This recommendation reflects a risk–benefit assessment based on several key considerations: (a) vaccinating at 32–36 weeks minimizes the risk associated with extremely preterm and very preterm birth, which carry substantial morbidity and mortality; (b) the MATISSE study demonstrated overall vaccine efficacy, with a subgroup analysis of individuals vaccinated at 32–36 weeks showing a similar point estimate of efficacy; (c) in contrast, the 24–28-week vaccination subgroup exhibited a lower point estimate of efficacy; (d) safety analyses indicated that a higher proportion of preterm births occurred in individuals vaccinated before 32 weeks; and (e) safety data further demonstrated a smaller difference in preterm birth incidence—0.5% compared with the overall 1.0%—among those vaccinated at 32–36 weeks [74]. Importantly, these considerations reflect regulatory caution rather than definitive evidence that earlier vaccination is less effective. The FDA’s recommendation prioritizes safety margins related to preterm birth while acknowledging that immunogenicity is adequate across gestational windows.

In the United Kingdom, Abrysvo^®^ is recommended from 28 weeks of gestation and may be administered up to delivery. Vaccination at or shortly after 28 weeks is preferred to optimize maternal antibody production and transplacental transfer, including for infants born prematurely. In the MATISSE trial, a nonsignificant imbalance in preterm births was observed between the vaccine and placebo groups, with no temporal association. No safety signal was detected in high-income countries; the imbalance was primarily observed in upper-middle-income countries, such as South Africa. Within 1 month of immunization, preterm birth rates were comparable between groups (2.1% vs. 1.9%). Vaccination later in pregnancy, including off-label administration after 36 weeks, may still provide some infant protection, as effective antibody transfer can occur within approximately 2 weeks [56]. The UK recommendation reflects a balance between maximizing antibody transfer (which increases with gestational age) and ensuring adequate time for antibody maturation before delivery [75].

Maternal immunization can result in the presence of antibodies in breast milk, potentially providing mucosal protection as infants ingest secretory IgA and IgG [88]. Although direct clinical evidence measuring Abrysvo-induced RSV antibodies in human breast milk is currently limited, this mechanism is biologically plausible and has been observed with other maternal vaccines. However, the clinical relevance of breast milk-derived antibodies for RSV prevention remains uncertain, as protective effects have not been demonstrated in randomized trials. Ongoing and planned studies, such as the NIH-sponsored PROMISE immunology study, are designed to quantify the magnitude and durability of RSV-specific antibodies in breast milk and infant serum following maternal vaccination, potentially clarifying the contribution of breastfeeding to infant RSV protection. Further research is needed to determine the clinical relevance of breast milk-derived antibodies, as their protective effect against RSV has not yet been established in randomized trials.

International differences in recommendations regarding repeat maternal RSVpreF vaccination reflect uncertainties in the durability of vaccine-induced antibody responses, the potential for immune interference in subsequent pregnancies, and the limited evidence base on safety and immunogenicity following repeated dosing. Current data suggest that antibody titers wane substantially within months after vaccination, but whether repeated immunization yields comparable transplacental transfer efficiency or altered IgG subclass distribution remains unknown.

### 5.5. Postmarketing Safety Data Regarding RSVpreF Vaccination (Abrysvo^®^)

#### 5.5.1. Preterm Birth

The first postmarketing safety data on pregnancy outcomes following RSVpreF (Abrysvo^®^) vaccination at 32–36 weeks’ gestation were reported by Son et al. [89]. In a cohort derived from electronic health records at two New York City hospitals (September 2023–January 2024), 1026 of 2973 pregnant individuals received RSVpreF, most commonly at 34 weeks’ gestation. Vaccination was not associated with an increased risk of preterm birth (5.9% vs. 6.7% in unvaccinated individuals), and no differences were observed in other obstetric or neonatal outcomes, including neonatal intensive care unit admission, jaundice/hyperbilirubinemia, hypoglycemia, or sepsis [89]. The generalizability of these findings is limited by the urban, institution-specific population and a preterm birth rate lower than the national average (6–7% vs. 10–11%). Moreover, outcomes were not stratified by preterm birth subtype, precluding assessment of potential differences between spontaneous and indicated preterm births [89].

During the 2024–2025 French immunization campaign, the safety of the RSVpreF maternal vaccine was evaluated in a nationwide cohort. Among 24,891 vaccinated pregnant women matched to unvaccinated controls, the median gestational age at vaccination was 34.7 weeks. Preterm birth rates were similar between groups (4.1% vs. 4.3%; incidence rate ratio [IRR] 0.97, 95% CI 0.89–1.06), and no increased risks were observed for delivery shortly after vaccination or for major obstetric or neonatal outcomes. Subgroup analyses indicated a nonsignificant elevation in preterm birth among women vaccinated at ≤32 weeks and a significant association in those also receiving influenza vaccination (IRR 1.25, 95% CI 1.02–1.53) [90]. Postmarketing evidence from the United States or the United Kingdom demonstrates comparable safety findings [91,92,93,94].

Overall, current postmarketing data do not indicate an increased risk of preterm birth associated with RSVpreF vaccination. However, subgroup signals—particularly among individuals vaccinated earlier in gestation or coadministered with influenza vaccine—require continued monitoring, as observational studies may be influenced by residual confounding, exposure misclassification and incomplete adjustment for clinical risk factors. These findings should therefore be interpreted cautiously and do not establish a causal relationship between RSVpreF vaccination and preterm birth.

#### 5.5.2. Hypertensive Disorders of Pregnancy

In the MATISSE trial, an observed imbalance in pre-eclampsia among vaccinated pregnant individuals prompted additional safety considerations. As a result, the generalizability of these trial findings to the broader pregnant population required ongoing monitoring and further evaluation using postmarketing surveillance data.

Postmarketing evidence has produced mixed results. In a cohort from TriNetX, a real-time US-federated health research network based on electronic health records, risk ratios and 95% CIs were calculated by comparing 6387 vaccinated pregnant women with matched unvaccinated controls [91]. Matching in the TriNetX analysis accounted for maternal age, demographics, healthcare utilization, comorbidities, medications, assisted reproductive technology use, multifetal gestation, parity, history of pregnancy complications, social factors, and body mass index. This study found no increased risk of gestational hypertensive disorders associated with RSVpreF vaccination [91]. Similarly, a statewide retrospective cohort study in Utah reported no increased risk of HDPs when comparing 2733 vaccinated and 21,480 unvaccinated pregnant women [92]. A cross-sectional study at a tertiary maternal hospital in London likewise found no difference in HDP frequency between 173 vaccinated and 738 unvaccinated pregnant women [93]. In addition, a nationwide cohort study during the 2024–2025 French immunization campaign reported no increased risk of pre-eclampsia (weighted IRR, 1.02; 95% CI, 0.85–1.22) (Table 4) [90].

In contrast, an analysis by Son et al. suggested a possible elevated risk of HDPs, particularly gestational hypertension [89]. However, the potential inclusion of early-onset cases (20–33 weeks’ gestation) raises concerns about immortal time bias. Immortal time bias occurs when outcomes that arise before an individual becomes eligible for vaccination are incorrectly attributed to the vaccinated group. Because early-onset HDP can occur before 32 weeks—prior to eligibility for RSVpreF—misclassification may artificially inflate risk estimates in observational datasets.

Furthermore, the Advisory Committee on Immunization Practices (ACIP) reported data from the 2023–2024 RSV season indicating a small but statistically significant increase in HDP risk associated with RSVpreF vaccination [94]. Importantly, no differences in HDP severity were observed between vaccinated and unvaccinated women, as reflected by comparable rates of cesarean delivery, postpartum hospital admission, and length of hospital stay [94]. The ACIP also noted that these findings could have been affected by residual confounding, incomplete adjustment for clinical risk factors, or outcome misclassification (Table 4) [94].

While Table 4 summarizes studies reporting both null associations and potential signals of increased hypertensive disorders of pregnancy (HDPs), the apparent heterogeneity in findings can be largely attributed to methodological differences across studies. Randomized controlled trial data, such as those from the MATISSE trial, offer the highest internal validity and did not demonstrate an increased risk of HDP following maternal RSVpreF vaccination. In contrast, observational studies suggesting a possible elevation in risk frequently relied on electronic health record or administrative datasets that are inherently vulnerable to residual confounding, exposure and outcome misclassification, and immortal time bias—particularly when early-onset HDP occurring prior to vaccine eligibility is not appropriately excluded from the vaccinated cohort.

Studies reporting no association (e.g., TriNetX, the Utah statewide cohort, the UK tertiary hospital cohort, and the French nationwide cohort) generally incorporated more comprehensive adjustment for maternal comorbidities, parity, socioeconomic factors, and healthcare utilization, thereby mitigating confounding to a greater extent. Conversely, studies indicating elevated risk often lacked granular clinical information or did not fully account for the temporal relationship between vaccination and HDP onset, which may have led to inflated risk estimates.

Taken together, a critical appraisal of the available evidence suggests that the inconsistent signals across studies are more plausibly explained by methodological limitations than by a true vaccine-related effect. For clinicians and policymakers, the most robust evidence—derived from randomized trials and rigorously adjusted observational analyses—does not support a causal association between maternal RSVpreF vaccination and hypertensive disorders of pregnancy. Continued postmarketing surveillance remains warranted, but the current evidence base supports the overall safety of RSVpreF with respect to HDP.

### 5.6. Real-World Effectiveness of RSVpreF Vaccination

A multicenter, retrospective, test-negative case–control study (BERNI study) was conducted during the 2024 RSV season in Argentina, the first country to implement a national maternal immunization program against RSV in infants. Of 633 infants hospitalized with LRTI, 505 (286 cases and 219 controls) met eligibility criteria for the primary vaccine effectiveness (VE) analysis. Among these, 51 (18%) cases and 109 (50%) controls were born to individuals who had received RSVpreF during pregnancy. VE against RSV-associated LRTI leading to hospitalization was estimated at 78.6% (95% CI, 62.1–87.9) from birth to 3 months of age and 71.3% (95% CI, 53.3–82.0) from birth to 6 months of age. VE against RSV-associated severe LRTI resulting in hospitalization was 76.9% (95% CI, 45.0–90.3) through 6 months of age. Notably, three in-hospital deaths attributable to RSV occurred, all among infants whose mothers had not received RSVpreF during pregnancy. The key strengths of the study include its test-negative design, which helps reduce biases related to care-seeking and testing practices, and the inclusion of hospitals across multiple regions of Argentina, enhancing geographic representativeness. Limitations include the potential for unmeasured confounding and temporal confounding related to vaccine uptake and RSV circulation patterns [95]. As with all test-negative designs, VE estimates may be influenced by differential healthcare-seeking behavior, misclassification of illness etiology, and residual confounding that cannot be fully eliminated despite adjustment.

A multicenter, prospective test-negative study conducted in the United Kingdom evaluated the effectiveness of maternal RSV vaccination against hospitalization for RSV-associated LRTI in infants during the 2024–2025 epidemic season (September 2024–January 2025). Overall, VE was 57.7% (95% CI, 28.2–75.1). Among infants born at least 14 days after maternal vaccination, effectiveness increased to 72.4% (95% CI, 47.8–85.4). The median age (interquartile range) of infants in the RSV case group (n = 391) and control group (n = 146) was 1.63 months (0.94–2.26) and 1.41 months (0.77–2.03), respectively. The proportion of full-term infants was 89% in the case group and 85% in the control group [96]. Differences in epidemic timing, healthcare utilization, and RSV testing practices across regions may partly explain variability in VE estimates between the UK and Argentina, rather than reflecting true biological differences in vaccine performance.

A retrospective nested case–control study in Scotland (August 2024–March 2025) assessed the real-world effectiveness of maternal RSVpreF vaccination among all singleton live births. Of 27,565 pregnancies, 13,842 women (50.2%) received the vaccine, with 92.1% immunized more than 14 days before delivery. The analysis included 354 infants hospitalized with RSV-related LRTI and 3511 matched controls. Optimal maternal vaccination (>14 days before delivery) occurred in 12.1% of cases versus 43.2% of controls, while suboptimal vaccination rates were similar between groups. Median gestational age at vaccination was approximately 30–31 weeks. Maternal RSVpreF vaccination demonstrated high adjusted effectiveness against RSV-associated LRTI hospitalization (82.2%; 95% CI, 75.1–87.3), which remained high in both preterm infants (89.9%) and term infants (81.5%). Sensitivity analysis using a matched cohort design yielded consistent results (81.0%; 95% CI, 68.6–88.5) [97]. The Scottish study benefits from comprehensive national data linkage, but as with all observational designs, VE estimates may still be affected by residual confounding, particularly related to maternal health status, socioeconomic factors, and timing of vaccination relative to RSV circulation.

Taken together, real-world effectiveness studies across Argentina, the United Kingdom, and Scotland demonstrate consistently high protection against RSV-associated hospitalization, despite differences in study design, population characteristics, epidemic timing, and healthcare utilization. Variability in VE estimates likely reflect contextual factors rather than true differences in biological effectiveness.

### 5.7. Comparative Effectiveness and Methodological Considerations

A recent study directly comparing maternal RSVpreF vaccination with nirsevimab highlighted imbalances in baseline characteristics—such as infant age distribution and living environment—between the two groups in France. From a causal inference standpoint, these differences introduce the possibility of residual confounding that may not be fully accounted for by statistical adjustments [98]. Because maternal vaccination and monoclonal antibody administration differ fundamentally in timing, mechanism of action, and target populations, direct comparisons between them are inherently susceptible to confounding by indication and selection bias.

These observed imbalances likely reflect the distinct mechanisms of action and delivery methods of the two preventive strategies. Maternal immunization confers indirect protection via transplacental transfer of maternal antibodies, while monoclonal antibody products provide direct protection through infant administration. These fundamental differences can introduce confounding by indication or selection biases in real-world comparative studies.

Furthermore, the temporal dynamics of immune protection differ between interventions. In infants born to Abrysvo-vaccinated mothers, antibody concentrations are highest at birth and gradually decline, whereas nirsevimab administration elevates RSV-specific antibody levels that remain sustained over a defined period. Such differences in antibody kinetics may create time-varying effects on RSV hospitalization risk, complicating causal interpretation in comparative effectiveness analyses. The French nationwide comparative study implemented a rigorous two-step confounding control strategy, combining exact matching on key perinatal variables with inverse probability of treatment weighting using an extensive set of maternal, infant, socioeconomic, and healthcare access covariates. Post-weighting balance diagnostics demonstrated excellent covariate balance, with standardized mean differences approaching zero across measured characteristics. However, even with advanced analytic techniques, residual confounding and time-dependent biases cannot be fully excluded in observational comparative studies. Differences in timing of exposure, eligibility for vaccination versus monoclonal antibody administration, and evolving RSV circulation patterns may introduce unmeasured sources of bias that affect comparative estimates. For these reasons, comparative effectiveness estimates should be interpreted as associations rather than causal effects, even when covariate balance diagnostics appear excellent. Randomized head-to-head trials would be required to establish true comparative efficacy. In this context, the recent JAMA comparative effectiveness study directly comparing maternal RSVpreF vaccination with nirsevimab is notable for its rigorous handling of time-related biases. Eligibility assessment, exposure assignment, and follow-up initiation were all aligned at maternity discharge, ensuring that both groups entered observation at a clearly defined and comparable time zero. The investigators also conducted lagged sensitivity analyses to account for incubation periods and symptom-onset windows, thereby further reducing the risk of immortal time bias and strengthening the temporal validity of their estimates [98]. From a policy perspective, maternal RSVpreF vaccination and nirsevimab offer complementary but distinct advantages. Maternal vaccination provides protection from birth and may enhance equity in settings with limited access to pediatric care, whereas nirsevimab offers predictable antibody levels independent of maternal factors and may provide longer-lasting protection. Accordingly, policy decisions should consider the differing mechanisms, implementation pathways, and population-level goals of each intervention rather than relying on observational comparative estimates to infer superiority.

### 5.8. Population-Level Impact

A test-negative case–control study conducted in the United States evaluated the population-level impact of maternal RSVpreF vaccination and nirsevimab as preventive strategies against RSV disease. Between October 2024 and April 2025, 5029 children younger than 2 years who sought medical care for acute respiratory illness (ARI) were enrolled. The median age was 10 months (interquartile range, 5–16 months), and 2176 children (43.3%) were female [99].

Among newborns and infants younger than 6 months, maternal RSV vaccination was estimated to be 64% effective (95% CI, 37–79%) against medically attended RSV-associated ARI and 70% effective (95% CI, 37–86%) against RSV-associated hospitalization. In contrast, nirsevimab effectiveness against RSV-associated hospitalization was estimated at 81% (95% CI, 71–87%), with protection remaining evident 130–210 days after administration, during which effectiveness was estimated at 77% (95% CI, 42–92%) [99]. Because this study did not directly compare the two interventions within the same analytic framework, differences in VE should not be interpreted as evidence of superiority of one strategy over the other. Instead, the findings highlight that both interventions contribute meaningfully to reducing RSV-associated morbidity at the population level.

Hospitalizations decreased by roughly 41–51% among infants 0–11 months and 56–63% among those 0–2 months, demonstrating substantial public health impact. These population-level reductions reflect combined effects of maternal vaccination, monoclonal antibody uptake, and changes in RSV circulation patterns, making it difficult to isolate the contribution of any single intervention.

Accordingly, population-level impact estimates should be interpreted as reflecting the integrated effects of multiple preventive strategies operating simultaneously, rather than the isolated effect of maternal vaccination or nirsevimab alone.

## 6. Future Outlook and Challenges

### 6.1. Concerns Regarding Pathogenic Strains and Variants

RSV comprises two major subtypes, RSV-A and RSV-B, with the dominant subtype varying by epidemic year and geographic region. Monitoring variant strains has gained increasing importance [20]. One study reported changes in the phylogenetic structure of RSV-B lineages before and after the COVID-19 pandemic, revealing shifts in genetic diversity and selective pressure [20]. Certain mutations have been suggested to potentially impact binding sites for long-acting monoclonal antibodies (e.g., nirsevimab), highlighting the need for ongoing surveillance to detect the emergence of resistant strains [100]. Despite these concerns, circulating RSV demonstrates gradual antigenic evolution, predominantly in the G glycoprotein, over decades in response to immune pressure [100]. In contrast, the F protein exhibits only modest antigenic changes over similar timescales, resulting in minimal reductions in neutralization by human polyclonal serum antibodies. The mechanisms underlying this relative antigenic stability remain unclear but may involve limited mutational tolerance or the broad targeting of multiple F epitopes by polyclonal antibodies, such that individual amino acid substitutions confer little selective advantage. Despite this stability, RSV reinfections occur repeatedly throughout life, likely reflecting the gradual waning of F-directed neutralizing antibody titers [100].

Traditionally, RSV molecular epidemiological was primarily based on the G gene. However, with the advent of vaccines and monoclonal antibody therapies, mutations in the F gene—critical for antigenicity—have also gained importance. While the G protein is highly variable and mutates frequently, the F protein is relatively structurally stable and serves as the primary target of neutralizing antibodies. In 2024, the HRSV Genotyping Consensus Consortium proposed a standardized phylogenetic classification method incorporating both the G and F genes. This approach addresses the limitations of traditional methods based solely on genetic distance and allows for flexibility to accommodate partial genomic data. Currently, databases such as the Global Initiative on Sharing Avian Influenza Data catalog both F and G genes. Monitoring mutations in these genes provides essential insights for evaluating vaccine and antibody effectiveness and for the early detection of potentially resistant RSV strains [101]. Although current evidence suggests that the F protein remains relatively conserved, the increasing use of monoclonal antibodies and vaccines may exert new selective pressures. Continued genomic surveillance is therefore essential to detect emerging variants that could affect the performance of preventive interventions. Although the F protein has remained antigenically stable over decades, widespread use of vaccines and monoclonal antibodies may introduce selective pressures that could, over time, favor rare escape mutations. Should meaningful antigenic drift occur, updated vaccine formulations or periodic booster doses may be required to maintain protection; however, current evidence does not indicate widespread resistance, and observed mutations at antigenic site Ø and other epitopes remain rare.

### 6.2. RSV Infection Control Measures for Infants and Young Children in Different Countries

At its September 2024 meeting, the Strategic Advisory Group of Experts on Immunization (SAGE) reviewed strategies to prevent RSV infection in children. Recognizing the substantial global disease burden, the SAGE recommended that all countries consider introducing vaccines to prevent severe RSV disease [102]. In May 2025, the SAGE further issued a position paper advising that countries decide between maternal RSV vaccination and the use of long-acting monoclonal antibody products for infants, taking into account factors such as cost, cost-effectiveness, supply and access, anticipated vaccination coverage, and the feasibility of integrating these interventions within existing healthcare delivery systems [103].

However, strategies for preventing RSV infection in infants and young children differ widely across countries. Maternal RSV vaccination is recommended in the United States, the United Kingdom, France, Australia, Japan, and several other nations [75,76,79,80,83,84,87]. In contrast, Canada and Germany do not currently recommend maternal vaccination, citing insufficient evidence to support routine use [81,82,85,86]. These differences reflect national policy judgments regarding safety monitoring capacity, cost-effectiveness thresholds, and programmatic feasibility rather than differences in biological efficacy. National policies also vary regarding the optimal gestational window for vaccination and whether vaccination should be offered during every pregnancy (Table 3).

International strategies for administering long-acting monoclonal antibody products to infants also vary. France [84,104] and Germany [85,86] recommend providing prophylaxis to all infants during their first RSV season, whereas the United States and Australia limit administration to infants younger than 8 months. The United Kingdom employs a more targeted approach, offering prophylaxis only to infants at high risk of severe RSV disease [75]. These policy differences reflect variations in healthcare infrastructure, cost-effectiveness thresholds, supply constraints, and local epidemiology rather than differences in biological efficacy of the products themselves. As more real-world data accumulate, national recommendations may converge or diverge further depending on emerging evidence.

### 6.3. Revaccination During Subsequent Pregnancies

The US Centers for Disease Control and Prevention (CDC) currently does not recommend revaccination for women who received a maternal RSV vaccine in a prior pregnancy, citing insufficient evidence [105]. During the ACIP discussions, members highlighted the need for additional data before recommending revaccination, noting potential vaccine-related safety considerations—such as preterm birth and gestational hypertension—as well as the availability of an alternative prophylactic option, nirsevimab, for infants born in subsequent pregnancies.

In contrast, the United Kingdom recommends administering Abrysvo^®^ after 28 weeks of gestation during each pregnancy, regardless of the interpregnancy interval [75]. This approach aims to maximize transplacental antibody transfer and ensure optimal protection for each newborn. Safety data from Phase 1 and 2 clinical trials in healthy adults indicate that revaccination is well tolerated, with a safety profile comparable to that of the primary dose (Table 3) [106]. However, these early-phase data were not designed to evaluate pregnancy-specific safety outcomes, and evidence on repeated dosing during pregnancy remains limited.

In Japan, revaccination during subsequent pregnancies is allowed and currently practiced, although no formal national recommendation has been issued. Revaccination is not prohibited under existing regulatory approval, and clinicians may administer RSVpreF in multiple pregnancies when deemed appropriate. However, national guidance has not yet specified whether routine revaccination should be recommended for all pregnancies, and further evidence on durability of protection, safety, and cost-effectiveness will be important for future policy development. This approach reflects a precautionary stance in the context of limited evidence rather than a biological rationale for or against repeated vaccination.

Given the divergent recommendations across countries, additional research is needed to clarify the durability of maternal immunity, the optimal interval between doses, and the safety and effectiveness of repeated vaccination during pregnancy. Longitudinal studies evaluating antibody kinetics across multiple pregnancies, as well as postmarketing safety surveillance, will be essential to inform evidence-based revaccination policies.

### 6.4. Concurrent Administration with Other Vaccines

Maternal vaccination protects against several infectious diseases, including seasonal influenza, tetanus, pertussis, SARS-CoV-2, and RSV. However, coadministration of maternal vaccines has generally not been studied in clinical trials, so current recommendations are based on data from the administration of individual vaccines. As more vaccines are recommended during pregnancy, understanding the potential impact of concurrent administration—on immunogenicity, efficacy, and maternal and neonatal outcomes—becomes increasingly important [107].

National advisory bodies provide guidance on maternal RSVpreF vaccination and coadministration with other vaccines. The ACIP (US) permits RSVpreF vaccination at any time before or after other recommended vaccines—including Tdap, seasonal influenza, and COVID-19—allowing for administration at different anatomical sites on the same day [6]. Similarly, Canada’s National Advisory Committee on Immunization, Australia’s Technical Advisory Group on Immunization, and the UK Joint Committee on Vaccination and Immunization support coadministration or flexible timing relative to other vaccines [87,108]. In contrast, the French National Authority for Health recommends a 2-week interval between Tdap and RSVpreF administration during pregnancy [83]. This precautionary interval reflects limited empirical evidence on coadministration rather than demonstrated safety concerns or known immunologic interference. Given the limited empirical evidence, coadministration recommendations remain largely precautionary. Future immunogenicity and safety studies will be essential to determine whether simultaneous administration affects antibody responses, reactogenicity profiles, or maternal–fetal outcomes.

### 6.5. A Health Economic Perspective

Across multiple settings, economic evaluations suggest that maternal RSVpreF vaccination (Abrysvo^®^) can be cost-effective or even cost-saving, depending on vaccine price, modeling assumptions, and healthcare system parameters (Table 5).

In six European countries, analyses reported that year-round maternal vaccination was cost-effective in Italy [109].

In Canada, a combined strategy of maternal vaccination at CAD 195 and nirsevimab for high-risk infants at CAD 290 yielded a smaller budget impact than universal nirsevimab administration while achieving comparable mortality reduction [110].

In Spain, maternal vaccination was dominant—providing both better health outcomes and lower costs—when priced at EUR 166.5 [111].

Economic modeling from Canada and the United States highlights that the cost-effectiveness of maternal RSVpreF vaccination depends heavily on vaccine pricing, delivery strategy, and population targeting. In Canada, combining year-round maternal vaccination with nirsevimab for high-risk infants was cost-effective when nirsevimab exceeded CAD 110–190 and Abrysvo cost less than CAD 60–125 [112], although at current prices, targeted nirsevimab alone was more cost-effective [112].

In the United States, a model assuming a vaccine price of USD 295 indicated that maternal vaccination timed immediately before or at the start of the RSV season could be cost-effective when measured in quality-adjusted life years (QALYs) [113].

Across diverse international settings, cost-effectiveness analyses of maternal RSVpreF vaccination reveal substantial variability depending on vaccine pricing, target population, and local healthcare context. Threshold analyses suggest cost-effectiveness in Japan at prices below JPY 23,948 [114]; in Argentina below USD 74.46 [115]; in Mexico, cost-saving below MXN 1301 and cost-effective between MXN 2105 and 3715 [116]; and in Australia below AUD 120 (or AUD 64 without herd effects) [117]. Because these analyses rely on heterogeneous assumptions—including RSV incidence, hospitalization costs, vaccine uptake, and willingness-to-pay thresholds—the results are not directly comparable across countries. Local economic evaluations remain essential for policy decisions. Across settings, key drivers of cost-effectiveness include RSV seasonality, hospitalization costs, vaccine or monoclonal antibody pricing, coverage rates, and health-system capacity. These contextual factors largely explain the variability in cost-effectiveness thresholds across countries, rather than differences in biological efficacy.

## 7. RSV Monoclonal Antibodies

For infant protection against RSV-associated LRTI, the ACIP recommends two complementary strategies: maternal RSVpreF vaccination during pregnancy or administration of the long-acting monoclonal antibody nirsevimab. Nirsevimab is indicated primarily for infants whose mothers did not receive maternal RSV vaccination, or for those born <14 days after maternal immunization, when transplacental antibody transfer may be insufficient. Healthy infants aged ≥6 months generally do not require nirsevimab, except when they have underlying conditions that increase the risk of severe RSV disease [118].

Nirsevimab is a long-acting monoclonal antibody designed to prevent RSV-associated disease in infants. It binds the highly conserved antigenic site Ø on the RSV pre-F protein and exhibits neutralizing activity over 50-fold greater than palivizumab [119]. Three amino acid substitutions in its Fc region extend its half-life in vivo to roughly 71 days, enabling protection with a single dose rather than the five monthly doses required for palivizumab [119,120].

Clinical trials in preterm infants demonstrated that a single dose of nirsevimab reduced RSV–LRTI-related hospitalization by 78% [52]. In healthy term-born infants, two multicenter Phase 3 trials—MELODY and HARMONIE—reported reductions in RSV–LRTI-associated hospitalization of 62% and 83%, respectively, during the first 12 months of life [121,122]. Real-world evidence also supports high effectiveness, with a population-based cohort study reporting 88.7% effectiveness against RSV-associated hospitalization [123].

A recent French study compared outcomes between infants protected by maternal RSVpreF vaccination and those receiving nirsevimab. As discussed in Section 5.7, comparative analyses between these two strategies require careful interpretation due to differences in timing, mechanism of action, and potential confounding. The French study implemented exact matching and inverse probability weighting and achieved excellent covariate balance; however, residual confounding, time-dependent biases, and differences in eligibility windows cannot be fully excluded. Therefore, comparative estimates should be interpreted as associations rather than causal effects.

A test-negative case–control study conducted in the United States evaluated the population-level impact of maternal RSVpreF vaccination and nirsevimab. Maternal vaccination was estimated to reduce RSV-associated hospitalization by 70%, while nirsevimab demonstrated 81% effectiveness [99]. These findings highlight that both interventions contribute meaningfully to reducing RSV-associated morbidity at the population level, and differences in point estimates should not be interpreted as evidence of superiority. Taken together, real-world comparative effectiveness studies published in JAMA and JAMA Pediatrics—using distinct but complementary designs—have consistently suggested greater protection associated with nirsevimab than with maternal RSVpreF vaccination, with some analyses indicating more sustained effectiveness of nirsevimab over time. Although these observational comparisons remain subject to residual confounding and time-related biases, synthesizing their findings provides clinically relevant insight: in settings where both options are available, infant monoclonal antibody administration may confer higher and more durable protection in certain risk groups, whereas maternal vaccination offers earlier, pregnancy-based protection and programmatic advantages. Rather than merely listing individual study results, integrating these data helps clinicians and policymakers weigh trade-offs between strategies when formulating RSV prevention policies.

Clesrovimab is another long-acting monoclonal antibody targeting RSV by binding antigenic site IV of the F protein, present in both pre-F and post-F conformations [124]. In a randomized, placebo-controlled trial, clesrovimab reduced RSV-related LRTIs requiring medical intervention from 6.5% to 2.6% and RSV-related hospitalizations from 28 to 9 cases [125]. These results support its use as an additional preventive option, particularly for infants whose mothers did not receive maternal RSV vaccination [126]. Its distinct binding site compared with nirsevimab may limit resistance development, and non-weight-based dosing simplifies administration [127].

As additional monoclonal antibodies become available, comparative effectiveness, the durability of protection, and cost-effectiveness analyses will be essential to guide optimal integration with maternal vaccination programs. Importantly, policy decisions should consider the complementary roles of maternal immunization and infant monoclonal antibody prophylaxis rather than relying on observational comparisons to infer superiority.

## 8. Conclusions

RSV remains a leading cause of severe LRTI in early infancy, imposing a substantial and largely preventable burden on healthcare systems worldwide. Recent advances in structure-based vaccine design, particularly stabilization of the pre-F F protein, have enabled the successful development of effective preventive interventions and have fundamentally transformed the RSV prevention landscape.

Maternal immunization with the RSV prefusion F vaccine (RSVpreF, Abrysvo^®^) provides clinically meaningful protection against severe RSV-associated disease and hospitalization during the first few months of life, when infants are at greatest risk and direct vaccination is not feasible. Evidence from randomized trials and real-world effectiveness studies supports maternal vaccination as a viable population-level strategy, with gestational timing emerging as a key determinant of effectiveness. However, gestational timing findings are derived from exploratory subgroup analyses that were not powered for formal interaction testing and therefore should be interpreted cautiously. Additional studies are needed to clarify the optimal vaccination window. These findings have important implications for vaccine policy, underscoring the need for clear national recommendations on optimal vaccination windows, integration into existing maternal immunization platforms, and effective communication strategies to maximize coverage.

The concurrent availability of long-acting monoclonal antibodies for infants, such as nirsevimab, further expands policy options. Rather than representing competing interventions, maternal vaccination and infant passive immunization should be considered complementary tools that can be deployed flexibly according to local epidemiology, healthcare delivery capacity, supply constraints, and cost-effectiveness considerations. Direct comparisons between maternal vaccination and monoclonal antibodies are limited by confounding by indication, time-dependent biases, and differences in target populations. As such, comparative effectiveness estimates should be interpreted as associations rather than causal effects. Policy decisions will need to address eligibility criteria, prioritization of high-risk groups, and coordination between maternal and pediatric immunization programs to avoid duplication while ensuring protection of vulnerable infants.

Sustained implementation of RSV prevention strategies will require continued molecular surveillance to monitor viral evolution and its potential impacts on antigenicity, alongside robust postmarketing safety and effectiveness monitoring in pregnant populations. In addition, unresolved policy questions—including revaccination in subsequent pregnancies, coadministration with other maternal vaccines, and financing mechanisms—highlight the need for adaptive, evidence-based policy frameworks. International variation in revaccination policies, including countries that recommend vaccination during every pregnancy and others that adopt a more cautious approach, underscores the need for further evidence on the durability, safety, and cost-effectiveness of repeated maternal RSVpreF immunization.

In conclusion, maternal RSV vaccination represents a cornerstone intervention for reducing RSV-associated morbidity in early infancy. When incorporated into coherent immunization policies alongside infant monoclonal antibody prophylaxis and strengthened surveillance systems, it offers a realistic and scalable pathway to substantially reduce the global burden of RSV disease. Ensuring equitable access, particularly in low- and middle-income countries, remains a critical challenge and a priority for future global health efforts. For low- and middle-income countries, international financing mechanisms will play a critical role. Gavi, the Vaccine Alliance, supports vaccine introduction by subsidizing procurement costs, negotiating affordable pricing, and strengthening national immunization programs, thereby enabling equitable access to newly introduced vaccines such as RSVpreF. Sustainable implementation will require robust financing mechanisms, including national vaccine purchase programs, reimbursement frameworks, and—where applicable—support from international partners such as Gavi to ensure equitable access in low- and middle-income countries.

## Figures and Tables

**Table 1:** Efficacy of Maternal RSV PreF Vaccination.

	Main Analysis			Final Analysis
	Gestational Age at Vaccination (Weeks of Gestation)			
	24–36		32–36	24–36
Efficacy analysis period (days after birth)	0–90	0–180	0–180	0–180
Medically attended LRTI %	57.1	51.3	57.3	49.2
CI	^¶^ 14.7, 79.8	^†^ 29.4, 66.8	^§^ 29.8, 74.7	^§^ 31.4, 62.8
Medically attended sever LRTI %	81.8	69.4	76.5	70.0
CI	^¶^ 40.6, 96.3	^†^ 44.3, 84.1	^§^ 41.3, 92.1	^§^ 50.6, 82.5
RSV-associated hospitalization %	67.7	56.8	48.2	55.3
CI	^‡^ 15.9, 89.5	^‡^ 10.1, 80.7	^§^ −22.9, 79.6	^§^ 23.8, 74.6

CI: confidence interval, ^¶^ 99.5%, ^†^ 97.58%, ^‡^ 99.17%, ^§^ 95%.

**Table 2:** The preterm birth rate demonstrated in MATISSE trial.

Target Population	Preterm Birth Rate (%)		Risk Ratio	95% CI
	Vaccine	Placebo		
Overall	5.7	4.7	1.2	0.98–1.46
Vaccination timing				
24–28 week of gestation	6.8	6.6	1.03	0.73–1.46
28–32 week of gestation	6.8	4.8	1.43	1.02–2.02
≧32 week of gestation	4.3	3.7	1.16	0.83–1.63
Income level (country)				
High	5.0	5.0	1.0	0.79–1.28
Non-high	7.0	4.0	1.73	1.22–2.47
Upper-middle	7.5	4.2	1.8	1.25–2.60
Lower-middle	2.6	5.1	0.51	0.05–5.43
Low	3.1	2.1	1.48	0.25–8.69

Cited from Japan Institute for Health Security RSV maternal immunization vaccine and antibody products fact sheet. (https://id-info.jihs.go.jp/immunization/basics/facts-sheets/RS_20251022.pdf, accessed on 22 October 2025) CI: confidence interval.

**Table 3:** International recommendations for maternal RSVpreF vaccination.

Country/Region	Regulatory Authority	Approved Gestational Window	Recommended Window for Routine Use	Revaccination in Each Pregnancy	Key Rationale/Notes
United States	FDA/ACIP	32–36 weeks September–January	32–36 weeks	Not recommended	FDA selected 32–36 weeks to minimize theoretical risk of preterm birth; ACIP cites adequate immunogenicity and safety within this window.
United Kingdom	MHRA/JCVI	28 weeks to delivery Year round	≥28 weeks	Recommended	JCVI prioritizes maximizing antibody transfer; no safety signal in high-income settings; vaccination allowed up to delivery.
European Union (EMA)	EMA	24–36 weeks	Country-specific (most adopt 28–36 weeks)	Varies	EMA approval based on MATISSE data; individual EU states adjust timing based on local policy.
Japan	PMDA/MHLW	28–36 weeks Year round	24–36 weeks	Allowed and currently practiced; no formal national recommendation	Revaccination is not prohibited and is occurring in clinical practice, although no explicit national guidance has been issued.
Australia	ATAGI	28 weeks to delivery Year round	28–36 weeks	Recommended	ATAGI emphasizes maximizing antibody transfer and alignment with pertussis vaccination timing.
France	HAS	28–36 weeks September–January	28–36 weeks	Recommended	HAS supports routine maternal vaccination; also recommends universal infant nirsevimab.
Canada	NACI	Not recommended	Not applicable	Not applicable	NACI concluded evidence was insufficient for routine maternal vaccination; prioritizes infant nirsevimab.
Germany	STIKO	Not recommended	Not applicable	Not applicable	STIKO cites insufficient evidence and cost-effectiveness concerns; recommends infant nirsevimab.

Abbreviations: FDA, Food and Drug Administration; ACIP, Advisory Committee on Immunization Practices; MHRA, Medicines and Healthcare products Regulatory Agency; JCVI, Joint Committee on Vaccination and Immunisation; EMA, European Medicines Agency; PMDA, Pharmaceuticals and Medical Devices Agency; MHLW, Ministry of Health, Labour and Welfare; ATAGI, Australian Technical Advisory Group on Immunisation; HAS, Haute Autorité de Santé; NACI, National Advisory Committee on Immunization; STIKO, Standing Committee on Vaccination (Germany).

**Table 4:** Postmarketing surveillance data focused on HDP.

Country	Outcomes	RSV Vaccinated	Non-RSV Vaccinated	Risk Evaluation	Ref.
US	Pre-eclampsia or Eclampsia	1381/6387 (21.6%)	1424/6387 (22.3%)	RR (95% CI): 0.97 (0.91–1.04)	[91]
US (Utah)	HDP	267 /2733 (9.8%)	1959/21480 (9.1%)	aOR (95% CI): 1.03 (0.90–1.19)	[92]
				aHR (95% CI): 1.04 (0.91–1.18)	
UK	HDP	13/173 (7.5%)	45/738 (6.1%)	*p* = 0.607	[93]
France	Pre-eclampsia	267/24891 (1.1%)	255/24891 (1.0%)	wIRR (95% CI): 1.02 (0.85–1.22)	[90]
US	HDP	203/1011 (20.1%)	355/1962 (18.1%)	aOR (95% CI): 1.10 (0.90–1.35)	[89]
				HR (95% CI): 1.43 (1.16–1.77)	
US (ACIP)	Any HDP	2344/13474 (17%)	2056/13474 (15.3%)	aRR (95% CI): 1.09 (1.03–1.15)	[94]

RSV, respiratory syncytial virus; RR, risk ratio; aOR, adjusted odds ratio; aHR; adjusted hazard ratio; HDP, hypertensive disorders of pregnancy; wIRR, weighted incidence rate ratio; CI, confidence interval.

**Table 5:** The cost-effectiveness of RSVpreF (Abrysvo^®^).

Country	Intervention Policy	Control	Analytical Perspective	Product Price	Cost-Effectiveness	Threshold	Funding
6 European countries [109]	1. Year-round administration of Abrysvo		Healthcare System and Social Welfare System	assuming 50 euros per session	Italy: prefer		Pharmaceutical company
	2. Year-round administration of Nirsevimab				-		
	3. Seasonal Administration of Nirsevimab				Netherlands: good, Italy: prefer		
	4. Seasonal + catch-up Administration of Nirsevimab				UK, Finland, Denmark, Italy: prefer, Scotland: cutting cost		
Canada [110]	1. Nirsevimab for all infants		Healthcare System and Social Welfare System	Threshold Analysis	preferred from a social perspective when the price per session is ≤CAD 290.	CAD 50,000/QALY	
	2. Nirsevimab for high-risk infants only						
	3. Year-round administration of Abrysvo + Nirsevimab for high-risk infants				prefer when administering Abrysvo at CAD 195 and Nirsevimab at CAD 290, with mortality suppression achieved.		
Spanish [111]	Year-round administration of Abrysvo		Healthcare System	166.5 Euro	Dominant	25,000 Euro/QALY	Pharmaceutical company
Canada [112]	Nirsevimab: year-round/seasonal/seasonal+catuch-up	Nirsevimab for high-risk infants	Healthcare System and Social Welfare System	Threshold Analysis Abrysvo; CAD 230, Nirsevimab; CAD 952	preferred when administering Nirsevimab at CAD < 110–190	CAD 50,000, CAD 100,000/QALY	Public
	Nirsevimab for midle and high-risk infants; year-round/seasonal/seasonal+catuch-up				prefer when administering at CAD 27,891/QALY		
	Pregnant woman; year-round administration of Abrysvo						
	Year-round administration of Abrysvo for pregnant women + Nirsevimab for high-risk infants				preferred when administring Nirsevimab at CAD > 110–190 and Abrysvo at CAD < 60–125		
USA [113]	Year-round or seasonal administration of Abrysvo	Palivzumab for high-risk infants	Healthcare System and Social Welfare System	Abrysbo; $295	year-round; $396.28/QALY seasonal; $163.513/QALY	$100,000, 200,00, 500,000/QALY	Public
Japan [114]	Abrysvo for all pregnant women + palivizumab for high-risk infant	Palivizumab for high-risk infants	Payor’s perspective and Social Welfare System	Threshold Analysis	prefer when priced at ≤¥23,948.	¥5,000,000/QALY	Pharmaceutical company
Argentina [115]	Abrysvo for all pregnant women		Healthcare System	Threshold Analysis	prefer when priced at ≤$74.46.	$10,636/QALY	Pharmaceutical company
Mexcico [116]	Abrysvo for all pregnant women		Healthcare System	Threshold Analysis	cost-saving when priced at MXN ≤ 1301, prefer when priced at ≤MXN 2105 or 3715	Mexican GDP multiplied by 1 to 3 (1 GDP per capita is MXN 247,310)	Pharmaceutical company
Australia [117]	Abrysvo for all pregnant women		Healthcare System	Threshold Analysis	preferred when priced at <AUD 120, prefer if <AUD 64, when excluding the herd effect on other age groups.	AUD 50,000/QALY	Public

## Data Availability

No new data were created or analyzed. Data sharing is not applicable to this article.

## Abbreviations

The following abbreviations are used in this manuscript:

Abbreviation	Full term
ACIP	Advisory Committee on Immunization Practices
ACS	Advisory Committee Statement
aHR	Adjusted hazard ratio
aOR	Adjusted odds ratio
ARI	Acute respiratory illness
ATAGI	Australian Technical Advisory Group on Immunisation
Arexvy	GSK RSVpreF3 vaccine
Abrysvo	Pfizer RSVpreF vaccine
CI	Confidence interval
EMA	European Medicines Agency
FDA	Food and Drug Administration
Fc	Fragment crystallizable region
FcRn	Neonatal Fc receptor
Gavi	Global Alliance for Vaccines and Immunization
HAS	Haute Autorité de Santé
HDP	Hypertensive disorders of pregnancy
IASR	Infectious Agents Surveillance Report
IFN	Interferon
IPTW	Inverse probability of treatment weighting
JCVI	Joint Committee on Vaccination and Immunisation
LMIC	Low- and middle-income countries
LRTI	Lower respiratory tract illness
MATISSE	Maternal Immunization Study for Safety and Efficacy
MAVS	Mitochondrial antiviral signaling protein
MDA5	Melanoma differentiation-associated protein 5
MHLW	Ministry of Health, Labour and Welfare
mRNA-1345	Moderna RSV vaccine (mRNA platform)
mRESVIA	Moderna RSV vaccine (brand)
NS1	Nonstructural protein 1
NS2	Nonstructural protein 2
PMDA	Pharmaceuticals and Medical Devices Agency
pre-F	Prefusion F protein
post-F	Postfusion F protein
QALY	Quality-adjusted life year
RCT	Randomized controlled trial
RIG-I	Retinoic acid-inducible gene I
RSV	Respiratory syncytial virus
RSVpreF	RSV prefusion F protein vaccine
RSVpreF3	GSK prefusion F protein vaccine
SAGE	Strategic Advisory Group of Experts on Immunization
SARI	Severe Acute Respiratory Illness
SH	Small hydrophobic
TLR4	Toll-like receptor 4
VE	Vaccine effectiveness
wIRR	Weighted incidence rate ratio
